# Biomechanical analysis of a magnesium plantar plate prototype system for the first tarsometatarsal joint fusion: a cadaveric study

**DOI:** 10.1186/s13018-024-05208-7

**Published:** 2024-11-28

**Authors:** Peng Zhou, Marx Ribeiro, Johannes Greven, Maximilian Praster, Jan-Marten Seitz, Simon Habicht, Frank Hildebrand, Elizabeth R. Balmayor, Philipp Lichte

**Affiliations:** 1 Experimental Orthopaedics and Trauma Surgery, Department of Orthopaedics, Trauma and Reconstructive Surgery, University Hospital RWTH Aachen, Pauwelsstraße 30, 52074 Aachen, Germany; 2 Medical Magnesium GmbH, Philipsstraße 8, 52068 Aachen, Germany; 3 Department of Orthopaedics, Trauma and Reconstructive Surgery, University Hospital RWTH Aachen, Pauwelsstraße 30, 52074 Aachen, Germany

**Keywords:** Lapidus fusion, Magnesium alloy, Absorbable implants, Locking plate, Hallux valgus

## Abstract

**Background:**

Titanium plantar plates have proven successful in the fixation of the first tarsometatarsal arthrodesis (TMT). However, a second surgery is typically needed for implant removal, and potential adverse effects, carried by the conventional implantations, are not uncommon. The purpose of this study was to determine whether a novel magnesium-based plantar plate system provides similar fusion stability to a titanium-based plantar plate system under various loading conditions.

**Methods:**

Six matched-pair human cadaveric specimens underwent TMT fusions using either a magnesium plantar plate system prototype or a titanium plantar plate system. Specimens were cyclically loaded with a force ranging from 5 N to 50 N for 5,000 cycles, and displacement was recorded. Axial stiffness (N/mm) was calculated from load-displacement curves. Each specimen was loaded to failure at a rate of 5 mm/min, and the ultimate load was recorded.

**Results:**

No significant difference was found in the vertical displacement between Ti group and Mg group after 100 cycles (2.4 ± 1.0 mm vs. 1.3 ± 1.4 mm, *p* = 0.196), 500 cycles (3.3 ± 1.3 mm vs. 1.7 ± 1.7 mm, *p* = 0.142), 1,000 cycles (3.7 ± 1.5 mm vs. 1.9 ± 1.9 mm, *p* = 0.128), 2,500 cycles (4.2 ± 1.7 mm vs. 2.3 ± 2.2 mm, *p* = 0.172) and 5,000 cycles (4.5 ± 1.8 mm vs. 2.3 ± 3.3 mm, *p* = 0.125), Additionally, no significant differences were observed in initial stiffness (53.1 ± 19.2 N/mm vs. 82.2 ± 53.9 N/mm, *p* = 0.257), final stiffness (90.6 ± 48.9 N/mm vs. 120.0 ± 48.3 N/mm, *p* = 0.319), or maximum load-to-failure (259.8 ± 98.2 N vs. 323.9 ± 134.9 N, *p* = 0.369).

**Conclusions:**

Based on the performed biomechanical testing, the magnesium plantar plate system provides mechanical stability equivalent to the titanium plantar plate system in fixation for the first TMT joint fusion.

## Introduction

The first tarsometatarsal (TMT) joint arthrodesis was introduced by Albrecht and Truslow to treat metatarsus primus varus [1, 2]. Paul Lapidus later popularized the procedure in the 1930s, thus establishing the eponym for this technique [3]. Currently, TMT arthrodesis is widely used to manage moderate to severe hallux valgus, especially for cases involving hypermobility of the first TMT [4, 5].

The plate used for fixation of the first TMT arthrodesis is usually made of non-biodegradable materials, such as titanium (Ti) and Ti alloys. It provides several benefits, such as favorable mechanical properties and excellent biocompatibility [6]. The possible disadvantages include a potential stress-shielding effect, artifacts in postoperative imaging, and the necessity for metal removal in case of complications even years after surgery or at the patient’s request for material removal. According to a meta-analysis that included 16 studies, the combined hardware removal rate for the first TMT arthrodesis amounts to 8.9% [7]. In addition, studies have reported that non-biodegradable materials are associated with various complications such as interference with skeletal growth, pain, screw loosening, soft tissue complications, and metal-related infections [8].

Despite their biodegradability, previously used bioabsorbable materials for implants have some disadvantages, including low mechanical strength and undesirable tissue responses [9]. In this respect, magnesium (Mg)-based alloys offer an alternative as promising degradable biomaterials for orthopedic applications [10]. The use of Mg-based alloys avoids a second surgery for implant removal and reduces adverse effects induced by the implantation of permanent biomaterials [11, 12]. In addition, Mg-based alloys exhibit excellent biocompatibility, sufficient mechanical properties, and favorable osteostimulative properties [13–15]. For this reason, Mg‐based alloys are being widely explored for a broad range of orthopedic applications.

The purpose of the study is to compare the biomechanical performance of two plantar plate systems (PPS), i.e., Mg-PPS and Ti-PPS under in-vitro conditions, an approach that is most commonly used in clinical practice. We hypothesize that there is no significant difference in-vitro between the Ti and Mg plate systems for fixation of the first TMT arthrodesis.

## Materials and methods

### Specimens preparation

Six fresh, matched pairs of cadaveric lower legs of an average age of 65.2 ± 10.7 years (*n* = 2 males, *n* = 4 females) were used for this biomechanical study. The specimens were frozen immediately after dissection from the body and allowed to thaw before fixation placement and further biomechanical testing. The thawing of frozen specimens was conducted at room temperature for at least 10 h before testing. This study was performed with approval from the Institutional Review Board at University Hospital RWTH Aachen (EK 048 − 21).

**Fig. 1:**
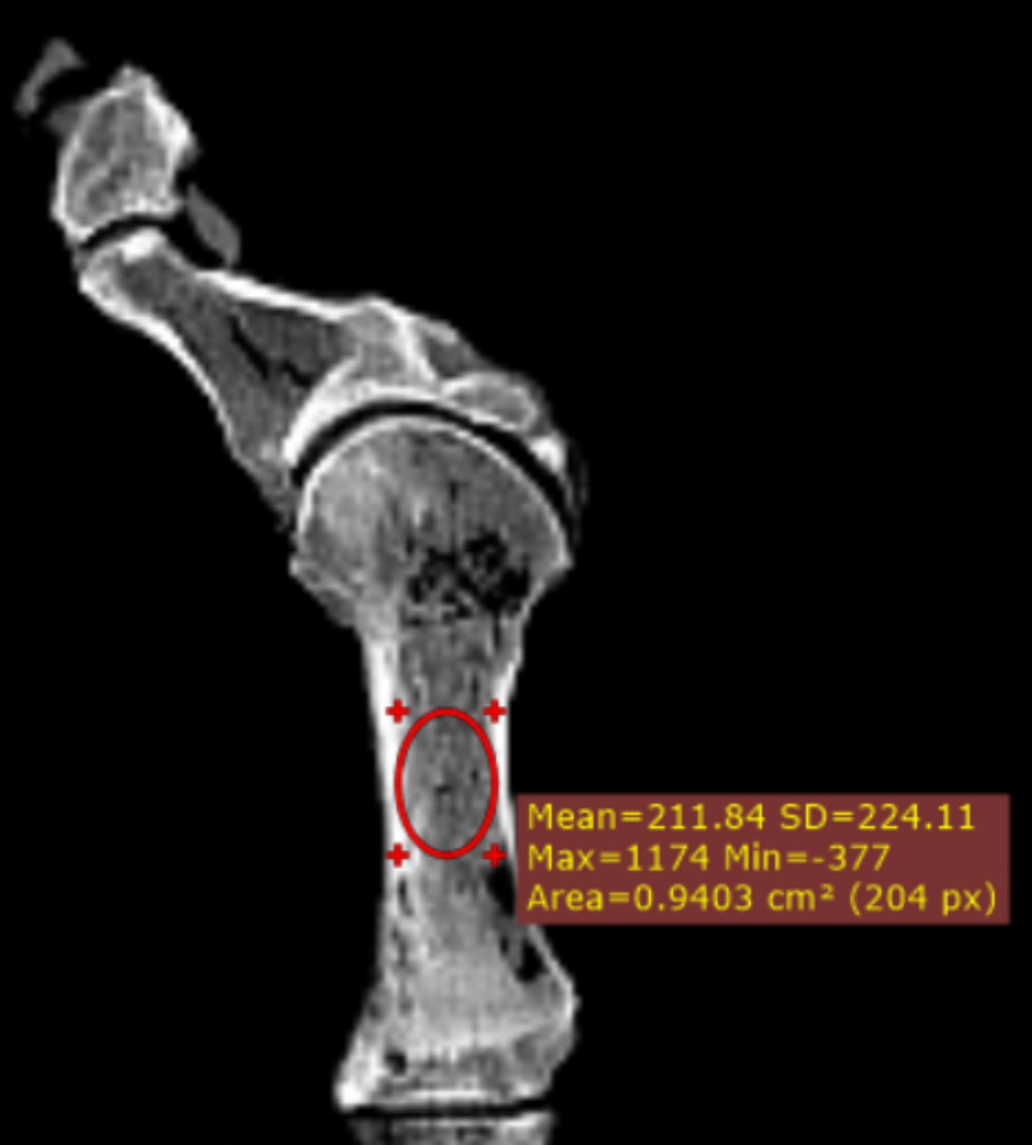
A representative sample of the specimens used in the study indicating the region of interest tool used to generate Hounsfield unit data from the first metatarsal computed tomography scan

Computed tomography was used to quantify bone density and to confirm that none of the specimens had any evidence of preexisting fracture or previous surgery. The bone density was accessed indirectly using mean Hounsfield units (HU) of the mid-shaft of the first metatarsals on computed tomography (Fig. 1), which are positively correlated with bone mineral density measurements obtained from dual X-ray [16].

**Fig. 2:**
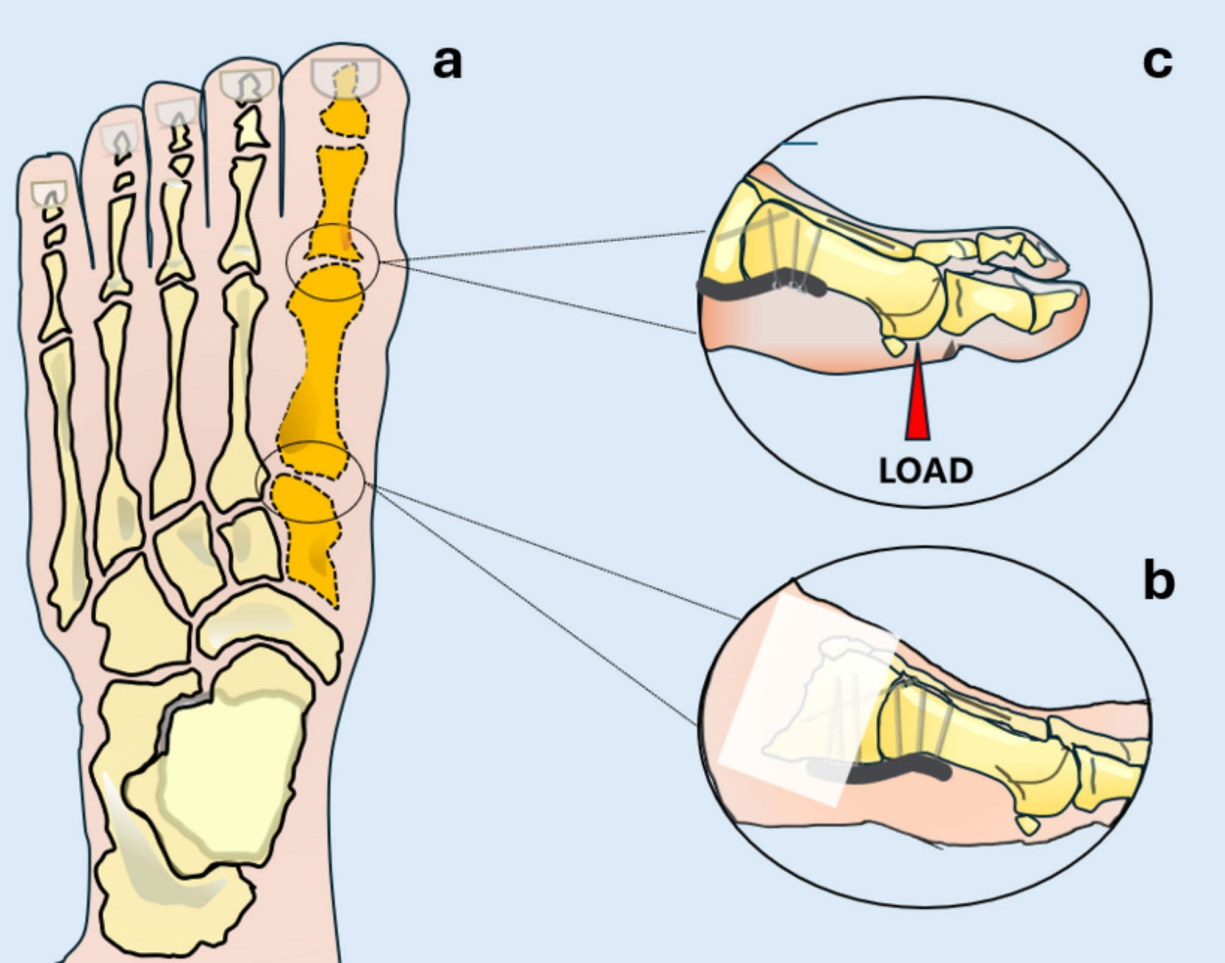
Schematic drawing of the biomechanical testing. (**a**) The highlighted sections (dark orange) indicate the first tarsometatarsal joint used for testing. (**b**) The medial cuneiform was embedded, with no connection between the plate and the embedding material. (**c**) The load was applied perpendicularly from the plantar to the dorsal surface of the first metatarsal

The specimens were cleaned, and all surrounding soft tissues were carefully eliminated. The specimens consisted of the first phalanges, the first metatarsal, and the medial cuneiform kept as a whole, preserving the joint capsules (Fig. 2a). Each pair was divided into two groups: one allocated to the Ti group and the other to the Mg group. All fixation procedures were performed by the same orthopaedic surgeon to ensure consistency.

### Instrumentation

**Fig. 3:**
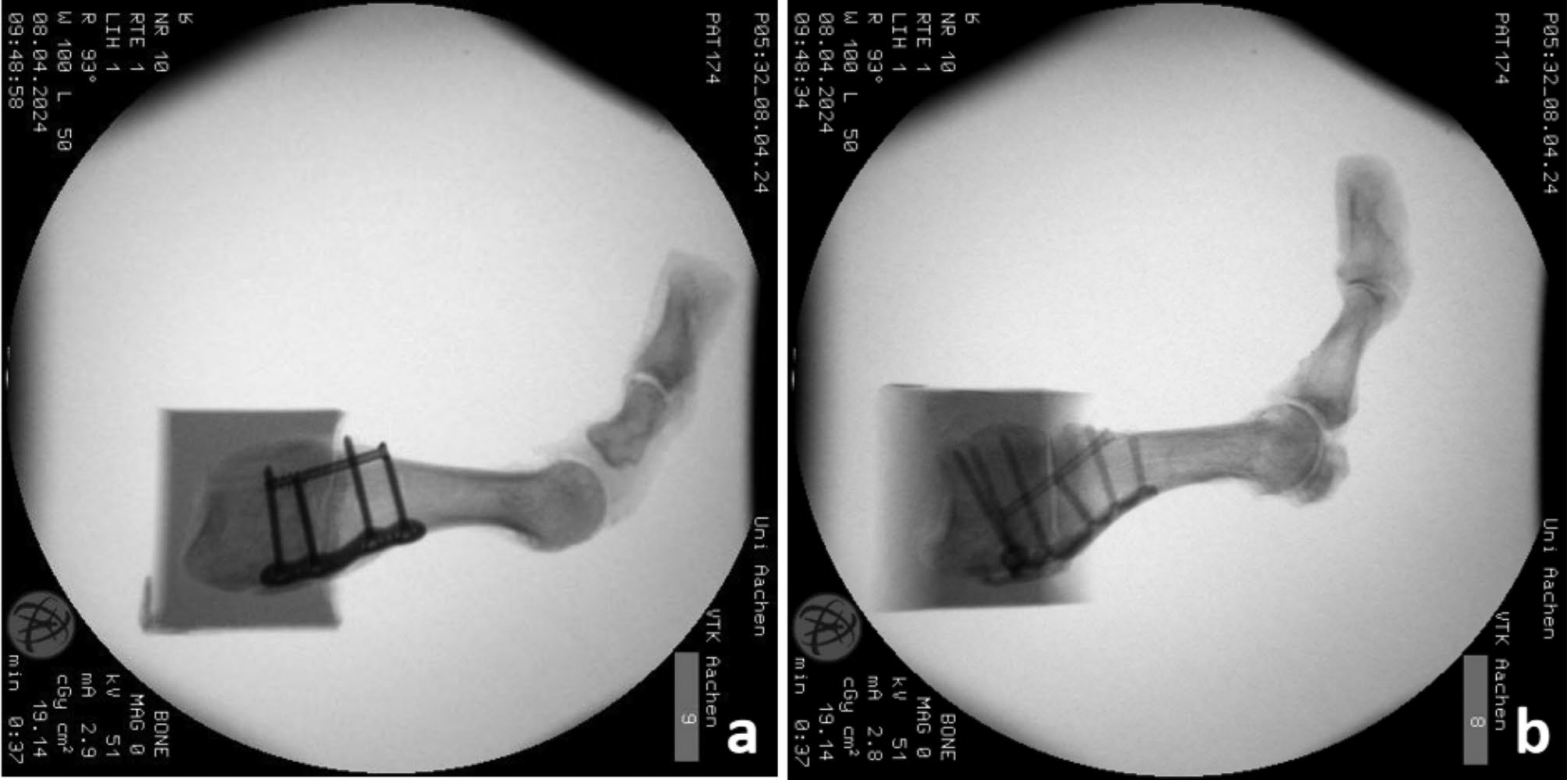
X-ray images of the two fixation groups. (**a**) Ti plantar-locking plate with crossed screw, (**b**) Mg plantar-locking plate with crossed compression screw

Based on the assigned groups, corresponding plates were used for fixation. In the Ti group, the specimens were fixed with the PEDUS-L Plantar Lapidus Plate (Dieter Marquardt Medizintechnik GmbH, Spaichingen, Germany), which is a Ti plantar locking plate with four holes for 2.7-mm angle-stable cortical screws, 39 mm long. Two proximal screws and two distal screws were inserted bicortically. The plate was then fixed on the plantar side (Fig. 3a).

In the Mg group, the specimens were fixed with a novel biodegradable Mg-PPS system (Medical Magnesium GmbH, Aachen, Germany), which consists of an Mg plantar locking plate with six holes for 2.7-mm angle-stable cortical screw prototypes. Three proximal screws and three distal screws were inserted bicortically at suitable lengths. For the Mg group, additionally, a 3.5 mm diameter compression screw (Medical Magnesium GmbH, Aachen, Germany) was inserted from the dorsal and distal side of the first metatarsal bone towards the plantar and proximal side of the cuneiform bone while a 4.0-mm diameter cannulated crossed screw was used in the Ti-group. All the Mg-based screws (Medical Magnesium GmbH, Aachen, Germany) were made of WE43MEO magnesium alloy and had a Plasma Electrolytic Oxidization (PEO) surface modification. Subsequently, the plate was securely fixed on the plantar side (Fig. 3b).

### Embedding

**Fig. 4:**
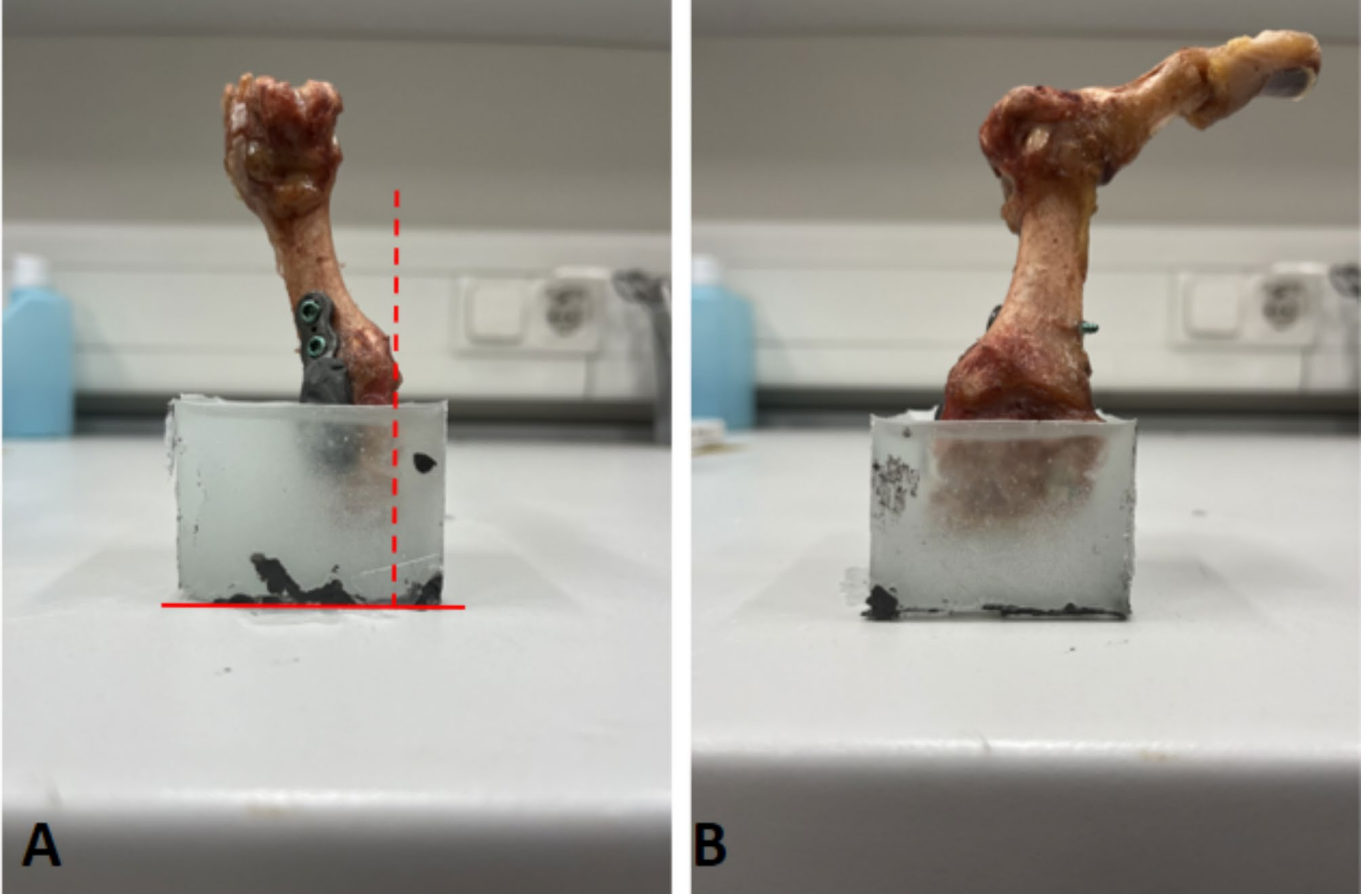
A representative image showing an embedded specimen fixed with the Ti plantar-locking plate. The lateral surface of the medial cuneiform is perpendicular to the horizontal plane. (**A**) Plantar view. (**B**) Lateral view

After instrumentation, the specimens were partially embedded in Technovit^®^ 4004 (Kulzer GmbH, Wehrheim, Germany) for fixation during testing (Fig. 2b). Care was taken to ensure that the Technovit^®^ 4004 does not apply additional pressure on the structure. A portion of the implant was covered with plasticine to prevent contact with the embedding material. During embedding, the lateral surface of the medial cuneiform was meticulously oriented perpendicular to the horizontal plane in a custom-designed potting fixture (Fig. 4).

### Mechanical testing

The biomechanical setup was established, and testing was performed according to the pretest and a previously published protocol [17]. Specimens were fixed in the test setup bottom-up in a dorso-plantar position. The test setup is comparable to a cantilever beam bending test with the fixation only in the bone, without contact with the PPS. The custom-built setup for compression load was assembled at a pneumatic testing machine (Dyna-Mess Prüfsysteme GmbH, Stolberg, Germany). The load was directed onto the distal end of the first metatarsal from the plantar to the dorsal surface, perpendicular to the ground (Fig. 2c).

**Fig. 5:**
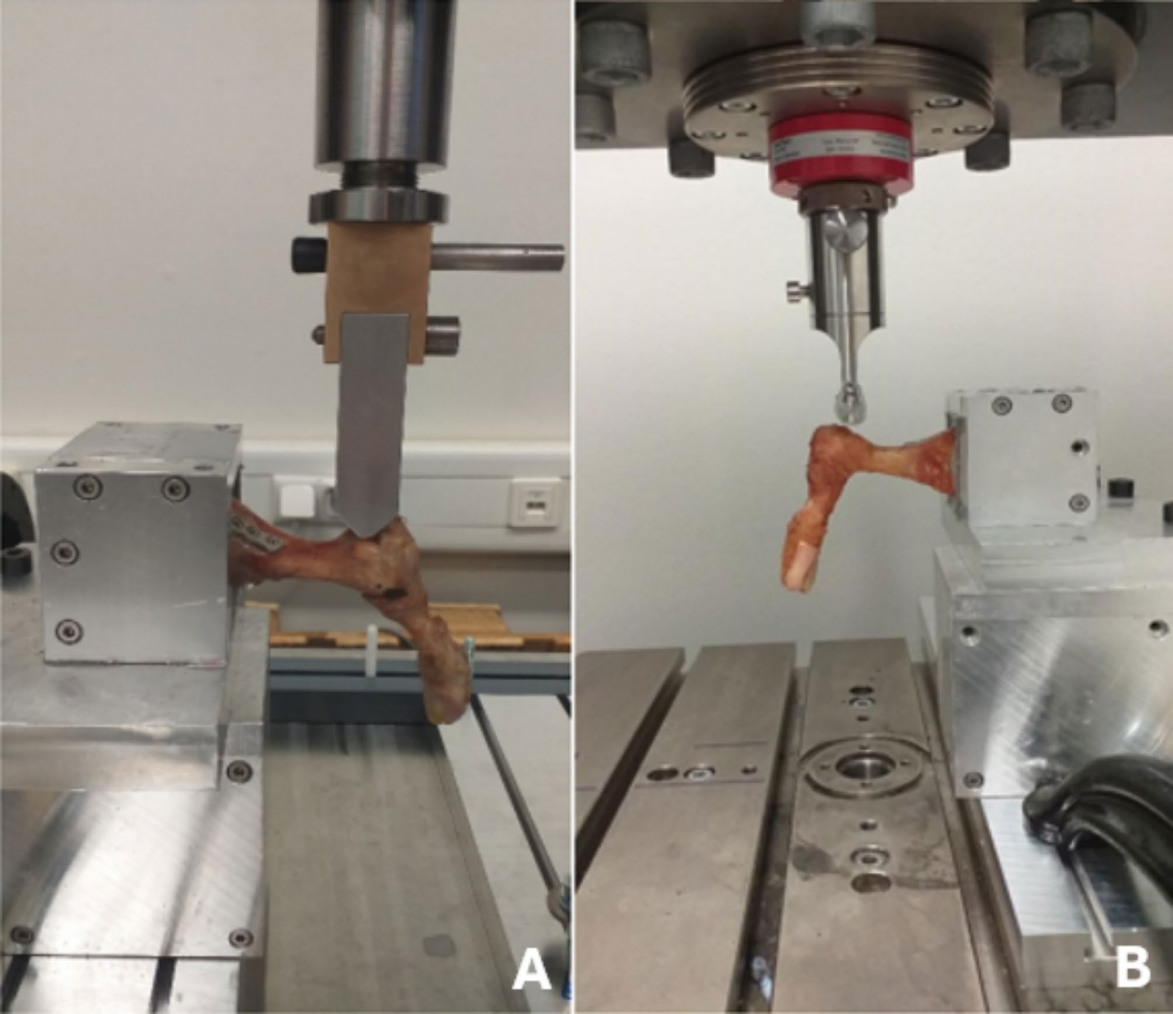
The views of two different loading test setups. (**A**) Cyclical loading test setup performed on a Dyna-Mess machine. (**B**) Load-to-failure test setup performed on a ZwickRoell universal testing machine

Each specimen was initially preloaded with 10 N. Subsequently, a cyclical load from 5 N to 50 N was applied at 0.5 Hz for 5,000 cycles [17]. This cyclical loading test aimed to simulate the postoperative partial weight bearing following the first TMT joint infusion [17].

After the cyclical loading test, the load-to-failure test was performed. Each specimen was loaded until fracturing with a speed of 5 mm/min (Fig. 5).

### Data treatment and statistical analysis

Prior to the test, sample sizes were calculated using G*Power software (G*Power 3.1.9.6) through a priori power analysis [18]. According to a previous study, a minimum clinically important displacement difference was selected to be 2.0 mm and a standard deviation of σ = 1.0 mm was assumed [19]. As the power and significance levels were set at *p* < 0.8 and *p* < 0.05, respectively, at least 6 specimens in each group were needed. The normality of data distribution was assessed and confirmed using the Shapiro–Wilk test (*p* > 0.05). The independent-sample t-test was applied to indicate significant differences between the Ti and Mg groups regarding mean HU values, displacement values in 100 cycles, 500 cycles, 1,000 cycles, 2,500 cycles and 5,000 cycles, initial and final stiffness, and maximum load to failure. Stiffness (K) was calculated as the slope of the force-displacement curve, defined using the following formula:

$$\:\text{K}=\frac{Fmax-Fmin}{dmax-dmin}$$

where *Fmax* and *Fmin* are the maximum and minimum force applied measured in Newtons, respectively, while *dmax* and *dmin* are the maximum and minimum displacement in the force actuator measured in millimeters, respectively. Additionally, for the load-to-failure test, Kaplan-Meier curves were generated from the survival data of the implants. Groups were compared using the log-rank test, the Tarone-Ware test, and the generalized Wilcoxon test. Statistical analysis was performed using SPSS software package (v.27, IBM SPSS, Armonk, NY, USA). The significance level was set at *p* < 0.05 for all statistical tests.

## Results

### Bone density assessment and cyclical loading tests

There were no statistically significant differences in the average HU value (256 ± 68 vs. 282 ± 52, *p* = 0.58) between Ti group and Mg group, regarding the first metatarsal. Of note, the specimens used in the study featured HU values that were in the range of those for the overall population (228 ± 71).

All six fixation constructs in both Ti and Mg groups survived the 5,000 cycles loading protocol without evidence of failure. Differences in displacement between the Ti and Mg specimens were not significantly different for each cycle group. The biomechanical and statistical results are summarized in Table 1.

**Table 1:** Vertical displacement and stiffness during cyclic loading compared between two groups

Description	Cycles	Ti group (*n* = 6)	Mg group (*n* = 6)	*p* value
Vertical displacement (mm)	100	2.4 ± 1.0	1.3 ± 1.4	0.196
	500	3.3 ± 1.3	1.7 ± 1.7	0.142
	1,000	3.7 ± 1.5	1.9 ± 1.9	0.128
	2,500	4.2 ± 1.7	2.3 ± 2.2	0.172
	5,000	4.5 ± 1.8	2.3 ± 3.3	0.125
Stiffness (N/mm)	0	53.1 ± 19.2	82.2 ± 53.9	0.257
	5,000	90.6 ± 48.9	120.0 ± 48.3	0.319

Ti, titanium-alloy; Mg, magnesium-alloy

### Load-to-failure tests

No statistical significance was concluded for the maximum load value when comparing the Ti group to the Mg group (*p* = 0.369) (Table 2).

**Table 2:** Maximum load-to-failure compared between two groups

Description	Ti group (*n* = 6)	Mg group (*n* = 6)	*p* value
Maximum load(N)	259.8 ± 98.2	323.9 ± 134.9	0.369
Variation ratio^a^	0.38	0.43	

Ti, titanium-alloy; Mg, magnesium-alloy

^a^The variation ratio is defined as the standard deviation divided by the mean value

**Fig. 6:**
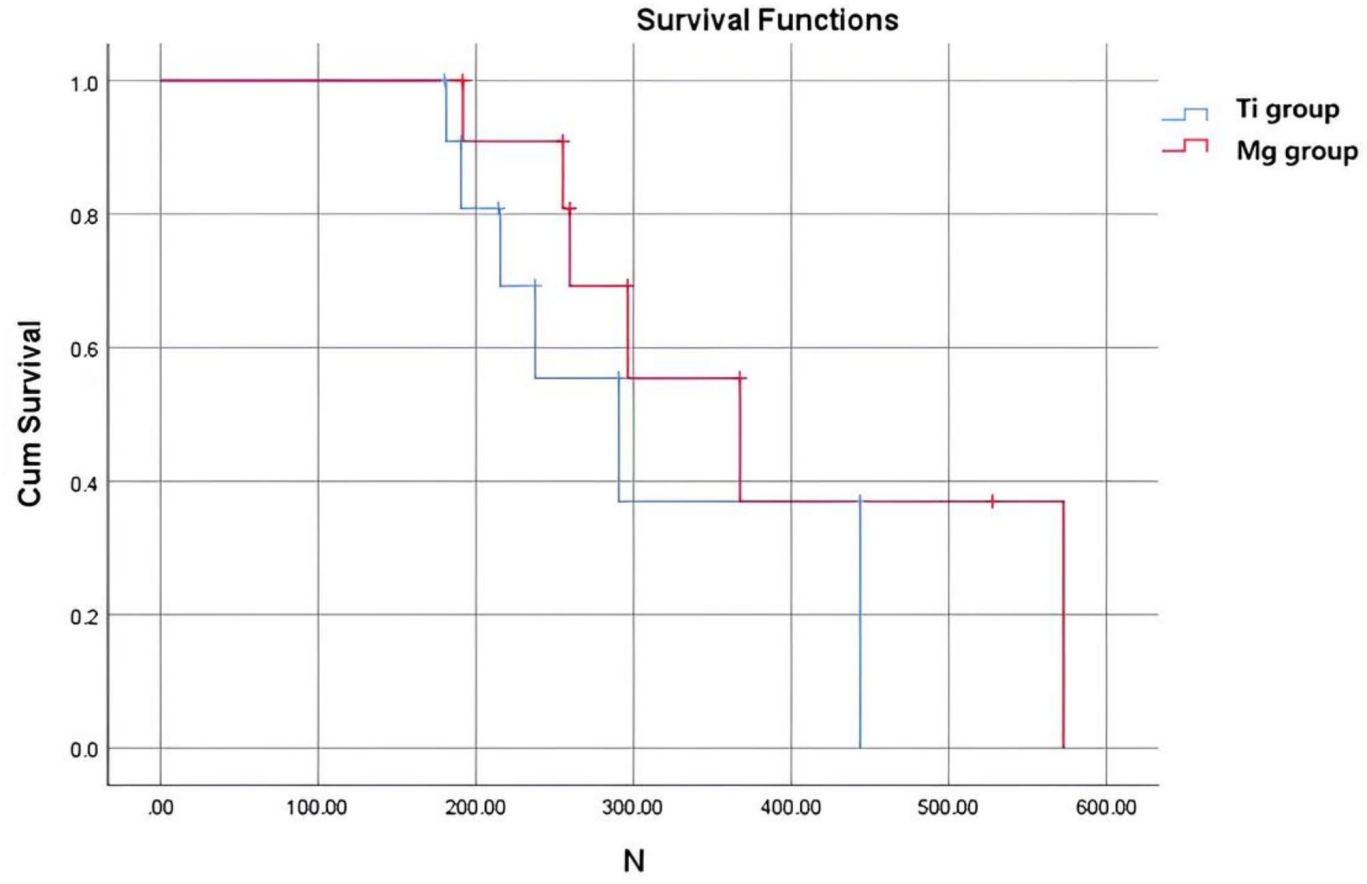
Survival analysis (Kaplan-Meier curves) of the Ti and Mg groups related to the applied force due to failure in Newton (N)

No significant difference was observed in the Kaplan-Meier curves (log-rank test *p* = 0.232, Tarone-Ware test *p* = 0.197, and the generalized Wilcoxon test *p* = 0.172) for the comparison between the two investigated groups, Ti and Mg fixation (Fig. 6).

## Discussion

The objective of this study was to compare Ti-PPS and Mg-PPS to determine the potential of a novel, biodegradable Mg-PPS for the fixation of TMT arthrodesis. To date, there is still a lack of evidence regarding the biomechanical properties of a Mg-PPS in fixation of TMT arthrodesis.

In the present study, there was no significant difference found in all performed evaluations, that is cyclic loading tests and load-to-failure tests between Ti and Mg-based plantar plate prototype systems. It should be noted that, the bone density was accessed for all specimens used in the study employing Hounsfield units. The values found were close to the average of the overall population [20], guaranteeing validity to the specimens used and obtained results.

The obtained mechanical testing results, confirm the hypothesis that a Mg-PPS and the currently commonly used Ti-PPS provide comparable initial mechanical stability and strength within the scope of this in-vitro study.

From a clinical perspective, previous studies have investigated the biomechanical properties of various hardware fixations for TMT arthrodesis such as crossed screws, dorsal-plantar / medial-plantar locking plates, and intramedullary locking devices [17, 21–24]. Among them, the advantages of the locking plates applied to the plantar aspect of the first TMT joint have been confirmed in in-vitro biomechanical models and clinical comparative studies [24]. Therefore, the locking plantar plate has been widely utilized in internal fixation for infusion of the first TMT joint.

Mg alloys were initially explored for musculoskeletal applications in the early 20th century [25] and have regained attention in recent decades. Medical implants made from magnesium-based alloys show significant potential for treating fractures due to their good biomechanical characteristics, which are close to those of cortical bone [11, 26]. Indeed, reports in the available literature regarding the in-vitro performance of Mg-based materials, is supportive to our findings. An in vivo study by Castellani et al. [27] reported that the biodegradable magnesium-alloy WE43 yielded higher maximum push-out force, ultimate shear strength, and energy absorption to failure than the Ti material used as control. Fischer et al. [28] compared the mechanical integrity between Mg mini-plates and Ti mini-plates in a sheep mandible model. The results of this in-vitro study showed no significant difference in the peak force at failure, stiffness, or force at vertical displacement of 1.0 mm between the Mg and Ti groups. By using a 3D finite element model of the human mandible, Orassi et al. [29] evaluated the biomechanical competence of Mg plates for mandibular fracture fixation. It was concluded that Mg devices showed a biomechanical performance similar to the clinically used Ti devices but, when compared to polylactic acid fixation devices, exhibited significantly higher primary stability.

Currently, orthopaedic implants made of Mg-based alloys are already in use in human applications, especially in foot and ankle surgery. These Mg-based implants, such as Herbert screws and pins, have shown good clinical effectiveness regarding the functional outcomes and biocompatibility [30–35]. Windhagen et al. [32] conducted a randomized controlled study to compare Mg screws and Ti screws for fixation of chevron osteotomy in patients with hallux valgus. The radiographic and clinical results reported by the authors showed that Mg-based screws are equivalent to Ti screws. Similarly, in a meta-analysis conducted by Fu et al. [36], radiographic and clinical results show that the Mg-based screws are equivalent to Ti screws for hallux valgus treatment in patients undergoing distal metatarsal osteotomy. These studies are aligned with our findings suggesting that Mg-based implants have great potential for clinical translation into human application, providing valuable insights and guidance for further advancement in this field.

The main drawback of using magnesium alloys as implants is their high corrosion rate in physiological environments [37]. Although there are several different methods available to enhance the corrosion resistance of magnesium implants, including alloying and coatings, there is currently a lack of research on these modified magnesium alloys in vivo [38].

This study has limitations. First, cadaveric in-vitro testing cannot accurately simulate the physiological loads of bony union in-vivo, including the effects of postoperative weight-bearing and rehabilitation practices. Second, the articular surfaces of the base of the first metatarsal and the distal aspect of the medial cuneiform were not denuded, which differs from clinical practice. This is due to the nature of the cadaveric specimens tested and the uncertainty as to whether the remaining bony surfaces would be able to interdigitate sufficiently. Third, the performed analysis did not account for the degradation process of the Mg plate. This aspect needs to be further investigated in in-vivo studies or in a long-term biomechanical experiment in the presence of a physiologically mimicking medium (e.g., simulated body fluid solution). Additionally, different screw locations and amounts of screws between the two groups may influence the experimental results. Finally, a relatively small number of specimens were used within this study due to limitations in obtaining cadaver pairs.

## Conclusion

In conclusion, our data demonstrate that the use of a Mg-based plantar plate system prototype can provide equivalent mechanical strength compared to a Ti-based plantar plate system in an in-vitro test setting. No significant differences were found between the Ti and Mg fixation groups regarding vertical displacement, stiffness, and maximum force, as determined by cyclic testing. From a biomechanical perspective, the results of this study are promising concerning the use of Mg plantar plate system in fixation for the first TMT joint fusion. Further, in-vivo evidence is required for a better understanding of the long-term effects of Mg plantar plate system on TMT fusion stability. This considering the biodegradability of magnesium as biomaterial.

## Data Availability

The data presented in this study are available on request from the corresponding author.

## Abbreviations

TMT: First tarsometatarsal arthrodesis
Ti: Titanium
Mg: Magnesium
PPS: Plantar plate system
HU: Hounsfield units
